# Disparities in oral cancer screening among Indian women aged 30–49: insights from a national survey

**DOI:** 10.3332/ecancer.2026.2096

**Published:** 2026-03-18

**Authors:** Navjot Kaur, Pritam Halder, Yuvaraj Krishnamoorthy, Gursimer Jeet, Garima Bhatt, Sathish Rajaa, Priyanka Sudhir, Rohit Sharma, Sarthak Tandon, Manish Gupta

**Affiliations:** 1 Otorhinolaryngology and Head Neck Surgery, Dr B R Ambedkar State Institute of Medical Sciences (AIMS, Mohali), Sector 56 A, SAS Nagar, Punjab 160055, India; 2 Department of Community Medicine and School of Public Health, Postgraduate Institute of Medical Education and Research, Chandigarh 160012, India; 3 Evidence Synthesis Unit, Partnership for Research, Opportunities, Planning, Upskilling and Leadership (PROPUL) Evidence, Chennai 600099, India; 4 Vancouver, V6M1T5, Canada; 5 Department of Health Sciences, University of York, YO10 5DD York, UK; 6 Department of Community Medicine, ESIC Medical College & PGIMSR, K.K. Nagar, Chennai 600078, India; 7 Sacramento, CA, USA; 8 Al Muhaidb Dental Clinic, Jazan 82724, Saudi Arabia; 9 Department of Oral and Maxillofacial Surgery, DMIHER (DU) Wardha 442001, Maharashtra, India; 10 Department of Radiation Oncology, Rajiv Gandhi Cancer Institute and Research Centre, Delhi 110085, India; a https://orcid.org/0000-0002-5563-724X; b https://orcid.org/0009-0008-2407-6807; c https://orcid.org/0000-0002-2319-0391; d https://orcid.org/0000-0002-2319-0391; e https://orcid.org/0000-0001-7784-308X

**Keywords:** inequalities in health, disparities, inaccessibility, oral cancer, national survey, women’s health

## Abstract

**Introduction:**

Oral cancer estimates are concerning in India, with inequalities in accessing screening services, especially in rural areas. Socioeconomic characteristics contribute to disparities in screening coverage. The current study estimates the coverage of self-reported screening, spatial patterns, differences in screening rates in urban and rural areas and determinants of screening among Indian women.

**Methodology:**

We analysed data from 348,882 women (30–49 years) participating in India’s fifth wave of the National Family Health Survey (NFHS-5). Self-reported oral cancer screening weighted coverage was estimated and compared per socio-demographic characteristics. Global and local spatial autocorrelation methods were applied to understand the spatial distribution of screening coverage, which was then depicted using choropleth maps. The differences in urban-rural screening were decomposed and determinants of screening were identified using the multivariable binary logistic regression. Analysis was done using Stata v17.0.

**Results:**

Overall, at the national level, self-reported screening coverage was 0.87%, with higher rates in urban areas (1.08%) compared to rural areas (0.77%). Screening uptake increased with age, socioeconomic status and education. Scheduled Tribes and the poorest quintile had the lowest rates. 348,882 participants were included in the final analysis after all exclusions. The uptake of oral cancer screening increased with an increase in wealth Index (Middle: adjusted odds ratio: 1.35; 95% CI: 1.07–1.70), Richer (1.43; 1.12–1.84), Richest (1.60; 1.20–2.13) and in obese women (1.28; 1.02–1.63). Meanwhile, women who belonged to the Muslim religion (0.68; 95% CI: 0.56–0.84), scheduled tribes (0.70; 95% CI: 0.53–0.84) and those who were illiterate (0.66; 0.51–0.85) had lower odds of screening uptake. Women from South Indian states (9.58; 95% CI: 7.60–12.07), West Indian states (3.81; 95% CI: 2.88–5.04), Central India (2.48; 95% CI: 1.95–3.14) and North-east Indian states (1.65; 95% CI: 1.20–2.27) had higher odds of oral cancer screening uptake compared to North Indian states. The urban-rural gap was 57.76% due to factor distribution and 42.24% due to differences in factor effects. Religion, caste, education and media exposure all significantly contributed to the gap.

**Conclusion:**

Screening uptake varied according to socio-economic status and region of the country. Significant disparities in oral cancer screening exist among urban and rural women, driven by socioeconomic factors. Enhancing healthcare access, education and media outreach in rural areas is essential to improving screening rates and reducing disparities.

## Introduction

With increasing life expectancy and changes in lifestyle patterns, the global burden of disease has shifted from infectious to chronic non-communicable diseases (NCDs), among which cancers have become increasingly prominent [1]. This epidemiological transition poses substantial challenges for health systems worldwide, with cancers contributing to nearly 19.8 million new cases and 9.74 million deaths annually and resulting in a loss of an estimated 250 million disability-adjusted life years [2]. Oral cancers affecting the mucosal surfaces of the lips, tongue, floor of the mouth and buccal mucosa have gained particular salience and rank among the top 20 most common cancers globally [3]. According to GLOBOCAN 2022, an estimated 377,713 new cases and 177,757 deaths are attributed to oral cancers globally, with South and Southeast Asia bearing the highest burden [4]. These estimates are overwhelming, particularly in resource-constrained countries. India alone contributes nearly one-third of the global oral cancer burden, accounting for 13%–16% of all cancers nationally [5, 6]. Most recent estimates from Indian population-based cancer registry data indicate that 63,671 women reported new oral cavity and pharyngeal cancer cases in 2024, corresponding to a crude incidence rate of 9.2 per 100,000 and a lifetime risk of about 1%, with mouth and tongue cancers forming the major subsites; additionally, these cancers contributed to approximately 37,418 deaths among women in 2024, reflecting a crude mortality rate of 5.4 per 100,000 [7].

Socioeconomic disparities play a critical role in the epidemiology of oral cancers in India, with the disease disproportionately affecting disadvantaged populations. Prominent risk factors such as widespread use of smokeless tobacco products like mishri, gutkha and tobacco paan, along with human papillomavirus infections, are strongly implicated in the high incidence of oral precancerous lesions and cancers [8]. Additionally, gender-specific risk behaviours and exposures, including increasing tobacco use among women and poor oral hygiene, are contributing to the rising burden among females, particularly in rural areas [9]. Presentation in late stages and inadequate access to diagnostic and treatment facilities, especially among females, lead to poor survival rates [10]. Early detection remains pivotal, as 5-year survival rates exceed 80% when diagnosed early, but drop to 20%–30% for late-stage diagnoses [11].

Recognising these challenges, the World Health Organisation and the Indian Council of Medical Research recommend integrating visual oral cancer screening into primary healthcare through both population-based and opportunistic approaches [12]. Randomised community-based trials in India have demonstrated that trained frontline health workers can effectively perform visual oral examinations, detect premalignant lesions and significantly reduce mortality from oral cancers [13]. Despite this evidence, national screening uptake remains extremely low. The fifth round of the National Family Health Survey (2019–21) report indicates that only about 1.2% of women aged 15–49 years have ever undergone oral cancer screening, with widespread urban-rural geographic disparities [14]. This is concerning, given that tobacco use and related risk factors remain more prevalent among rural women, as documented by recent rounds of the Global Adult Tobacco Survey [15–17]. Additional barriers such as lower health literacy, sociocultural norms, gendered restrictions on mobility and decision-making and disparities in primary healthcare access further impede screening uptake in rural settings [12, 18].

Although oral cancer incidence is higher among men, studying women separately is essential because they face distinct structural and gender-linked barriers that disproportionately limit screening uptake. Women often have lower autonomy, restricted mobility and reduced decision-making power regarding their health, contributing to delayed care-seeking and poorer outcomes. Moreover, India’s population-based NCD screening platforms, delivered largely through frontline workers and reproductive-health-aligned community systems, interact more consistently with women, making them an operational priority group for early detection. A female-focused analysis, therefore, facilitates a clearer understanding of gender-specific inequities and supports the development of tailored, equity-oriented interventions [19, 20]. Given these concerns, understanding the socio-demographic and geographic determinants of oral cancer screening uptake among women is essential. Women aged 30–49 years represent the priority population for oral cancer screening under India’s National Programme for Prevention and Control of NCDs, and fifth wave of the National Family Health Survey (NFHS-5) provides complete and nationally representative data for this age group. Therefore, the present study was done to examine the coverage of oral cancer screening in urban and rural areas stratified by socio-demographic characteristics, determine the spatial patterns and identify any regional variations, ascertain the factors contributing to urban-rural disparities and those influencing the likelihood of oral cancer screening separately among women aged 30–49 years, residing in urban, rural and overall Indian settings.

## Methodology

### Study design and data source

We re-analysed a dataset from the NFHS-5, which is freely available in the public domain. The NFHS, India’s version of the Demographic Health Survey, began in 1992–93, providing nationally representative, de-identified and state- and gender-disaggregated data on maternal, child and later men’s health for policy analysis [14, 21, 22].

### Study population

The NFHS prioritises maternal and child health, sampling 636,699 households with 724,115 women (15–49 years old) and 101,839 men (15–54 years old). Using multi-phase stratified cluster sampling, Census Enumeration Blocks and villages served as Primary Sampling Units, selected via the Probability Proportional to Size sampling technique [14]. The data were recorded electronically in local languages using different study tools. Specifically, the Women’s tool inquired about a broad range of subjects, including inquiries about screening for breast, cervical and oral cancer [14]. The Biomarker tool for women included measurements of height (in cm), weight (in kg), waist and hip circumference (in cm), haemoglobin (mg/dL), blood pressure (mm of hg) and random blood sugar measurements (mg/dL).

### Study sample

**Figure 1.**
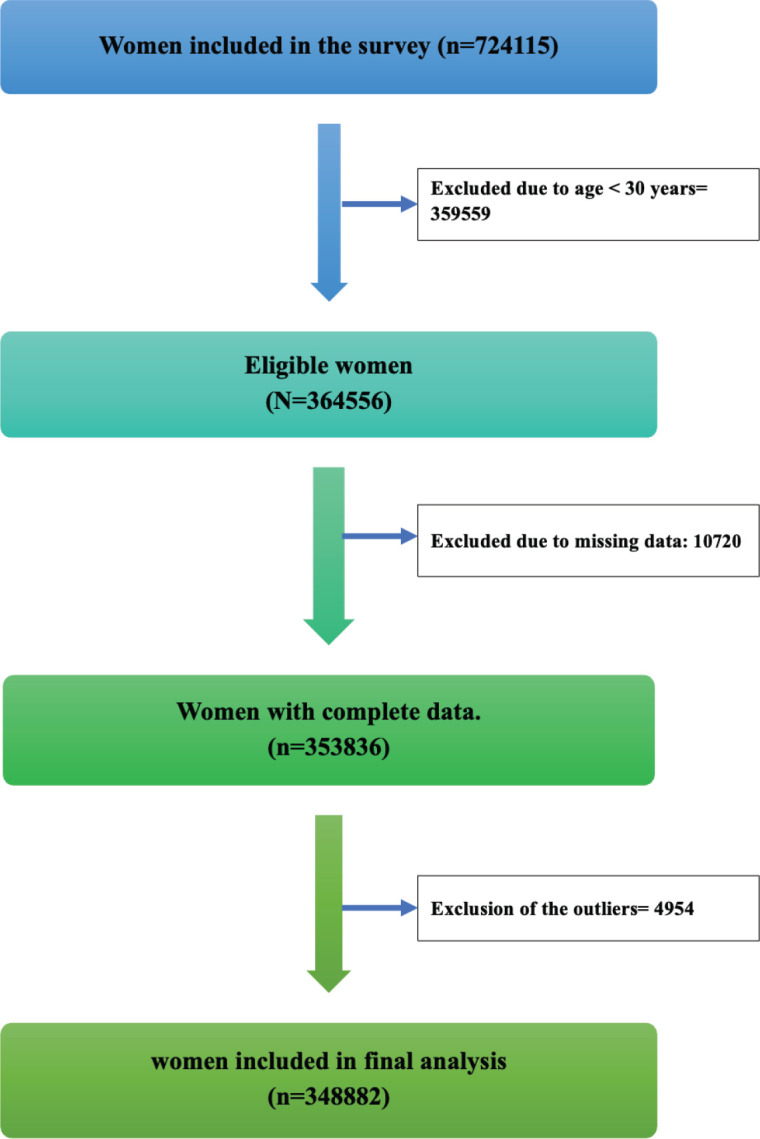
Sample selection process used in the study.

Of the 724,115 women who participated in the NFHS-5, we excluded women <30 years (*n* = 359,559). The 30–49-year age group was selected based on national screening guidelines, which recommend this age band for preventive cancer screenings among women [23]. We further excluded women with missing data (*n* = 10,720) and outliers (*n* = 4,954) using a complete case analysis to obtain a final sample of 348,882 women (Figure 1).

### Study variables

Self-reported oral cancer screening was our main dependent variable. The women were enquired using a direct question, ‘Have you *ever undergone a screening test for oral cancer*?’ and the responses were recorded dichotomously as yes or no. We selected independent variables based on a literature review and the scope of the current dataset and to reflect the PROGRESS-Plus domains – Place of residence, Caste, Religion, Education, Occupation (via wealth proxy) and Social capital (via media exposure) and ‘plus’ to identify other probable variables such as disease status or disability, enabling a structured examination of health inequities [20, 24–26]. The PROGRESS-Plus approach enables an intersectional analysis of the barriers faced by specific subgroups, such as rural women from Scheduled Tribes with low educational attainment and limited media exposure [27, 28]. Thus, we included age groups in years (30–34, 35–39, 40–44 and 45–49), religion (Hindu, Muslim, Christian and Others such as Sikh, Jain, Buddhist, Parsi, Jewish, No religion), Caste (scheduled caste (SC), scheduled tribe (ST), Other Backward Class (OBC) and others such as General Category/No caste/not SC/ST/OBC), education (illiterate, primary secondary and higher secondary), gender of head of household (male, female), marital status (married,and others which included never in union, widowed, divorced, separated, no longer living together), health insurance (yes/no), region of India (north, central, east, northeast, west and south) and Socio-economic status that was estimated using the wealth quintiles (poorest lowest 20% quintile ), poorer, middle, richer, richest (highest 20%-quintile 5) hat was derived from the wealth index. Other variables included the Body Mass Index (BMI), living with diabetes or hypertension, frequency of eating fruits, fried food and behavioural risk factors like tobacco, alcohol consumption, along with exposure to mass media. The variables were categorised as per our previous paper, which can be found elsewhere [24].

### Data analysis

The analysis plan is based on the author’s previously published paper assessing disparities in cervical cancer screening among the women of reproductive age group of India [24]. We conducted a bivariate analysis using STATA v17.0 to calculate the coverage of self-reported oral cancer screening across different independent variables, reporting weighted proportions and their 95% confidence intervals. The associations were tested using a Chi-squared test. Both global and local spatial autocorrelation methods were applied to understand the spatial distribution of screening coverage. Global analysis using Moran’s I, based on Queen’s first-order matrix, assessed the overall spatial pattern – whether coverage was clustered, dispersed or randomly distributed using GeoDa software version 1.14. A significant pseudo-*p* value (<0.05) from 999 permutations confirmed the presence of spatial autocorrelation, with positive values indicating clustering and negative values suggesting spatial outliers [29]. However, global measures alone do not reveal where these patterns occur. Therefore, Local Indicators of Spatial Association and Getis-Ord and Local Geary statistics were employed to identify specific clusters, including hotspots, cold spots and outliers. Differences in disparities were assessed using the Blinder–Oaxaca decomposition technique, which disaggregates the total disparity into two components: one attributable to differences in observed characteristics (e.g. education, media exposure, wealth and caste) and another attributable to differences in the impact or returns of these characteristics. The latter reflects structural inequities or systemic barriers that persist even when demographic and socioeconomic profiles are similar [30]. This framing enables a more nuanced equity analysis, aligning with the PROGRESS-Plus framework [31]. Additionally, the determinants of oral cancer screening coverage were examined using multivariable binary logistic regression. Results were presented as adjusted odds ratios (aOR) with 95% confidence intervals and a *p*-value of <0.05 was considered statistically significant.

## Results

**Figure 2.**
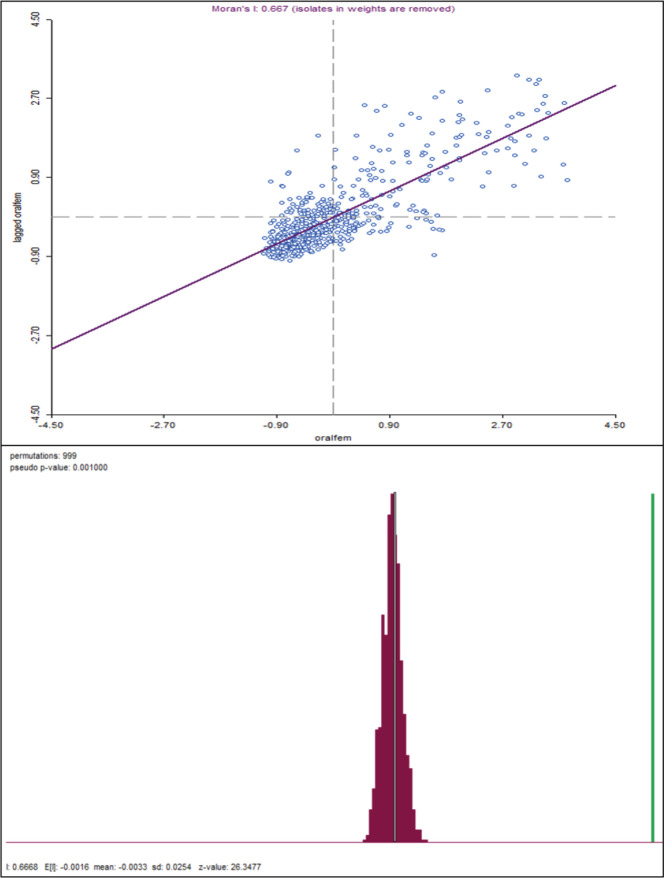
Moran’s I scatter plot showing spatial autocorrelation of oral cancer screening coverage among Indian women aged 30–49 years, along with permutation-based significance testing (999 permutations).

**Figure 3.**
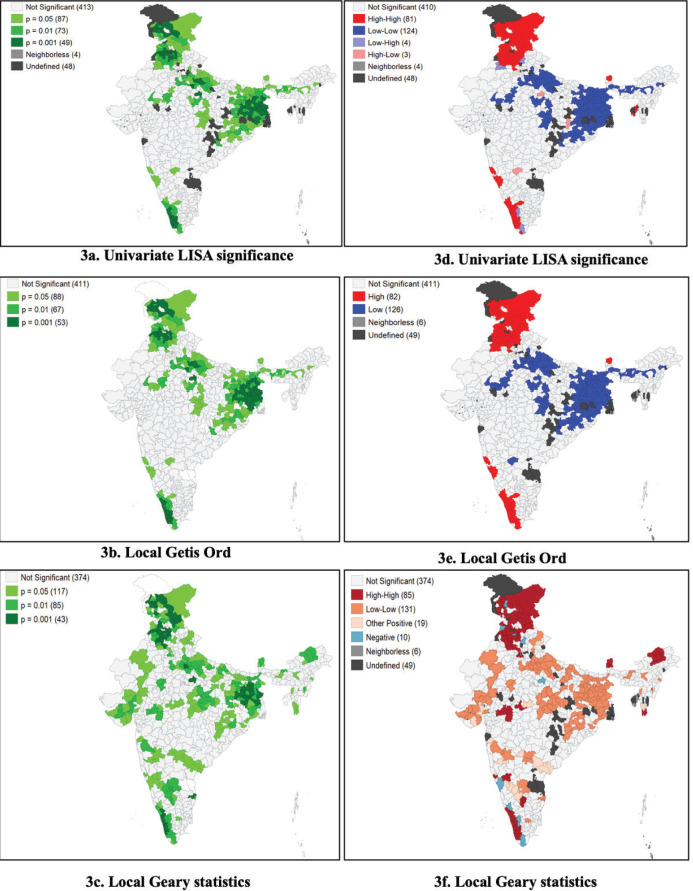
Spatial clustering of the oral cancer screening coverage (3a–c) and geographical clustering of hotspots and cold spots (3d–f).

Of the 348,882 women analysed, the national coverage of self-reported screening for oral cancers was 0.87% (95% CI: 0.84–0.90). The rural areas (0.77%; 95% CI: 0.74–0.80) exhibited lower coverage than urban areas (1.08%; 95% CI: 1.04–1.12). Table 1 provides an elaboration of the coverage proportions by socio-demographic characteristics. The screening rates increase with age and peak in the 45- to 49-year-old age group. Urban women in this age group (1.34%), Christians (2.34% overall) and the richest urban quintile (1.18%) had a higher screening rate. Scheduled Tribes (0.44% overall) and the poorest quintile had the lowest screening rates (0.37% in urban and rural areas). Education levels were positively associated with screening rates. Female-headed households had higher screening rates, particularly in urban areas (1.24%). Married women also had higher screening rates, with urban areas showing a rate of 1.08% compared to 0.75% in rural areas. Health insurance significantly increased screening rates, especially in urban areas (1.62% versus 1.17% in rural areas). Women living with obesity (High BMI) had the highest screening rates (1.23%), with urban obese women having a rate of 1.31% compared to 1.17% in rural areas. Women with diabetes and hypertension also had higher screening rates, with urban women showing higher rates (2.75% and 1.37%, respectively) compared to rural women (1.80% and 1.08%, respectively). Behavioural factors such as daily fruit consumption were associated with higher screening rates, especially in urban areas (1.24% compared to 1.03% in rural areas). Tobacco users had lower screening rates, particularly in rural areas (0.39%). Smokers had lower screening rates overall, with rural smokers having a rate of 0.43%. Alcohol consumers had lower screening rates, with rural consumers showing a rate of 0.54%. Women exposed to media had significantly higher screening rates, particularly in urban areas (1.13% compared to 0.90% in rural areas) (Supplementary Table S1). Regional differences were evident, with Southern India having the highest screening rates (2.31% overall) and urban areas showing slightly lower rates (2.29%) compared to rural areas (2.33%). The Northeast region had a low overall screening rate (0.33%), with urban areas exhibiting a higher rate (0.71%) compared to rural areas (0.23%). Overall, West Bengal had the lowest coverage (0.09%). The lowest coverage in the urban and rural areas was revealed in Uttarakhand and Lakshadweep, respectively. Andaman and Nicobar Islands had the highest coverage (10.32% each) in urban and rural areas (Supplementary Table S2). There were spatial disparities in coverage as confirmed by Moran’s I statistic (0.667) (Figure 2). Higher coverage proportions, also known as hotspots, were spatially clustered in states like Kerala, Punjab and Jammu & Kashmir. On the contrary, low coverage or cold spots depicted clustering in states from the Central, Eastern and Northeastern regions (Figure 3).

Table 2 depicts the results from the decomposition analysis conducted to estimate the contributions of various independent variables to the urban-rural differences in the screening coverage. The overall urban-rural difference was statistically significant (*p* = 0.317). Approximately 57.76% of the differences were significantly attributed to the endowment, while 42.24% were attributed to the coefficients of the independent variables. Among the factors that significantly explained the disparity, the age group 40–49 showed negligible effects in reducing the gap. The Muslim religion significantly contributed to reducing the gap from the urban perspective by 6.41%, while it diminished the gap from the rural perspective by 15.16%. Castes other than OBC, SC and ST also made a positive contribution, with an 11.87% increase from the urban perspective.

From the urban perspective, higher secondary education and health insurance reduced the gap by 9.17% and 5.91%, respectively. Among health-related factors, diabetes significantly contributed to widening the gap from the urban perspective by 3.57%. Among behavioural factors, daily consumption of fruits contributed to reducing the gap. Occasional fruit consumption widened the gap from the urban perspective and diminished it from the rural perspective. Tobacco positively contributed from the urban perspective by 2.22% and negatively from the rural perspective by −5.99%. Media exposure contributed to widening the gap from urban and rural perspectives by 3.87% and 58.62%, respectively. The South region had the most considerable positive contribution to the urban-rural gap, with a 33.73% increase from the urban perspective. The Eastern and Western regions made positive contributions, while the Central and Northeastern regions made negative contributions to the gap from an urban perspective.

**Figure 4.**
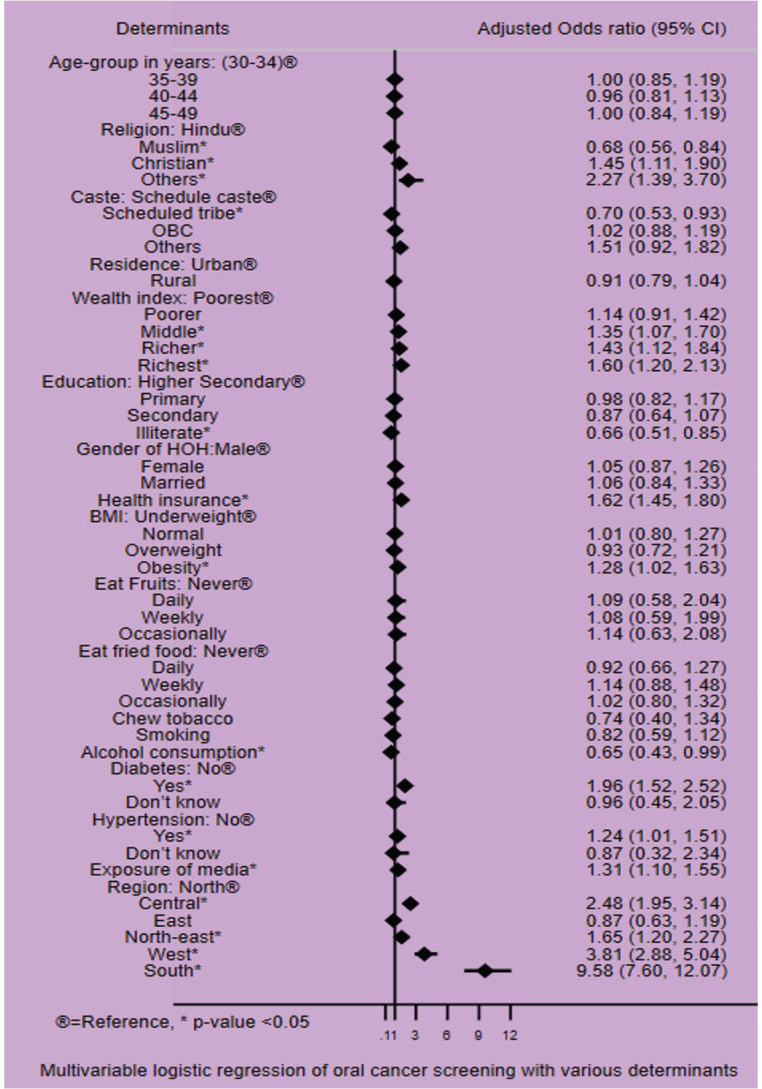
Determinants of oral cancer screening among Indian women participants (30–49 years) from the NFHS-5.

Figure 4 illustrates the socio-demographic determinants of oral cancer screening among Indian women. The likelihood of oral cancer screening coverage increased with the wealth index from middle to richest (Middle wealth index, aOR 1.35 (95% Confidence Interval: 1.07–1.70), Richer﻿ (1.43; 95% CI: 1.12–1.84), Richest (1.60; 95% CI: 1.20–2.13)) compared to poorest. Illiterate women underwent less screening uptake for oral cancer as compared with those who had higher secondary educational status (0.66; 95% CI: 0.51–0.85). Christian women had higher odds, whereas Muslim women had lower odds for uptake of oral cancer screening compared to Hindu women (Christian: 1.45; 95% CI: 1.11–1.90, Muslim: 0.68; 95% CI: 0.56–0.84). Women who belonged to the Scheduled tribes had lower odds for screening uptake (0.70; 95% CI: 0.53–0.84). Obese women had higher uptake for screening compared with underweight women (1.28; 95% CI: 1.02–1.63). Women living in Central, North-east, West and Southern India had higher odds of screening uptake compared with North India (Central: 2.48; 95% CI: 1.95–3.14, North-east:1.65; 95% CI: 1.20–2.27, West: 3.81; 95% CI: 2.88–5.04, South: 9.58; 95% CI: 7.60–12.07).

## Discussion

This study reveals disparities in self-reported oral cancer screening among Indian women aged 30–49 years, highlighting how structural and social health determinants shape access to preventive services. Using a nationally representative dataset and a PROGRESS-Plus informed lens, we found that factors such as place of residence, caster, education and socioeconomic status significantly shape screening behaviours, underscoring persistent inequities in India’s health system. The extremely low national average and persistent urban-rural divide in screening uptake reflect deeply entrenched barriers to preventive care services.

It is also important to interpret the low screening uptake in the context of the COVID-19 pandemic, as NFHS-5 fieldwork overlapped with multiple phases of nationwide lockdowns from March 2020 to April 2021 [32]. During this period, routine NCD services were widely disrupted due to mobility restrictions, repurposing of health workers for COVID duties, closure of outpatient services and fear of infection [33]. National reports indicate major declines in preventive and early-detection services during this period, suggesting that the NFHS-5 estimates reflect both true low coverage and temporary service interruptions.

Place of residence, a foundational PROGRESS variable, emerged as a critical driver of inequity. Rural women- particularly from marginalised communities- face limited availability of services, weaker health system infrastructure and sociocultural constraints that restrict access [34]. Despite the presence of recognised risk factors such as tobacco and alcohol use, overall screening uptake among Indian women remains extremely low, indicating that structural and gender-linked barriers outweigh individual risk profiles in shaping screening behavior [19, 20, 35]. Indian women commonly prioritise household responsibilities, childcare and family wellbeing over their own health needs, a pattern documented across chronic diseases and women’s cancers [36–38]. Studies consistently show that women delay or avoid preventive care due to limited autonomy, time constraints and dependence on family members for travel [39]. These gender-linked constraints likely contribute substantially to low oral cancer screening uptake.

Caste-based disparities, particularly for STs, persist as a marker of historical marginalisation. Women from tribal communities had the lowest screening rates (0.44%) with significantly lower odds of screening services uptake, even after adjusting for socioeconomic factors. These findings reinforce how caste operates not just as a social identity but as a determinant of health opportunity, intersecting with geography, gender and poverty to compound exclusion [40]. Previous studies also report similar findings [25]. Women belonging to the marginalised communities were reported to be 1.37 times more likely to lack access to healthcare services compared to men from the general category [41]. Religion, particularly among Muslim women, was associated with lower oral cancer screening uptake (aOR 0.68; 95% CI: 0.56–0.84), while Christians depicted the highest screening rates, followed by women following Hinduism. A systematic review by Kretzler *et al* [10] also reported that cancer screening practices vary among different religions. While cultural beliefs may partially explain these differences, structural and autonomy-related factors may also contribute, and contrasting evidence across studies warrants cautious interpretation [19, 25, 35].

Education emerged as a strong enabler of screening, reinforcing the link between health literacy and the agency in health-seeking behavior. Women with no education had significantly lower odds of screening (aOR 0.66 (95% CI: 0.51–0.85)), with a clear gradient in screening rates across education levels. Illiterate women, who form a large proportion of rural, tribal and poorer groups, are often excluded from health communication strategies [42]. Another plausible explanation for low uptake is the misinterpretation of the screening question among women with low literacy. Although NFHS-5 interviews were conducted in local languages, studies show that women often confuse routine dental or oral examinations with cancer screening. In settings with limited prior exposure to cancer-related services, the concept of ‘screening’ itself may be unfamiliar, leading to underreporting or inconsistent responses [43]. This challenge has been noted in other surveys involving cancer screening, where women frequently misrecognised the nature or purpose of screening tests [44]. Such misclassification and misunderstanding may therefore partly contribute to the very low prevalence of self-reported oral cancer screening observed in our study.

The wealth gradient was consistently associated with screening uptake, with the richest quintile nearly twice as likely to be screened as the poorest, findings consistent with earlier work [3, 8, 20, 45]. Economic marginalisation intersects with other PROGRESS categories, particularly rural residence, low education and minority status, compounding social disadvantage. Given the persistent social gradient, policymakers must prioritise intersectional targeting rather than siloed outreach to single groups [20, 25, 35]. The data also suggested that female-headed households were associated with higher screening rates. In urban settings, 1.24% of female-headed households underwent screening compared to 0.81% in rural areas. This trend aligns with earlier research indicating that female-headed households were more likely to participate in breast and cervical cancer screening programs [20]. However, a contrasting study reported higher screening rates in male-headed households, underscoring the contradictory evidence from some studies, which reflects the complexity of gendered household decision-making in healthcare utilisation [20]. Media exposure played a significant role in widening the disparities across the socio-economic gradient, with rural women less likely to benefit from health promotion activities and campaigns. Previous studies also highlight the role of exposure to mass media in substantially increasing screening rates, particularly in urban areas [46, 47]. These findings underscore the need for inclusive communication strategies that account for disparities in access to digital and traditional media channels. Studies such as Changkun *et al* [20] show that women with higher education were significantly more likely to undergo oral cancer screening compared to those with no formal education in urban (aOR 1.29) as well as rural areas (aOR 1.24). Overall, education consistently enhances health literacy and screening uptake, although some variability across studies suggests that contextual differences may exist.

Regional disparities identified in the current analysis further underscore the inequitable implementation of oral cancer screening programs. The Southern region reported the highest screening uptake among women, across urban and rural areas, suggesting that cancer screening programs in these states may have been integrated into the primary health system, possibly benefiting from prior successful health campaigns and community health worker engagement. The findings align with Sen *et al* [35] who reported higher compliance with breast and cervical cancer screening in Tamil Nadu and Kerala, states known for their higher literacy levels and relatively stronger public health infrastructures. In contrast, the northeast and eastern regions exhibited alarmingly low coverage, with rural areas particularly underserved. Geospatial analysis reaffirmed these patterns. A significant Moran’s *I* statistic of 0.667 indicated strong spatial clustering of screening coverage, with hotspots concentrated in Punjab, Jammu & Kashmir and Kerala, and cold spots identified in central, eastern and northeastern regions. These findings align with the analysis by Gopika *et al* [48], which noted the highest oral cancer screening uptake among women in the Andaman and Nicobar Islands (10.1%). These variations suggest that differences in programme intensity, community engagement and health-system readiness, rather than geography alone, drive regional performance [20]. However, the NFHS-5 lacks information on programme delivery, health-system readiness or contextual factors that might explain these differences, and we cannot determine why some regions perform better or worse. Future studies should examine these high- and low-performing settings to identify transferable lessons for strengthening oral cancer screening across India. Some states may have benefited from pilot initiatives, donor-supported programs or stronger health system capacity; however, such information is not captured in the NFHS dataset, which limits our ability to interpret regional variations [49, 50].

Women with obesity or with diabetes/hypertension had higher screening rates. While this differs from some international evidence linking obesity to lower screening, contextual differences in care-seeking and provider engagement may explain these patterns [51, 52]. A similar contrast was seen for tobacco users, who had lower screening rates, particularly in rural areas, and the results are comparable to sub-national studies specifically targeting oral cancer screening [53]. Despite being at higher risk, lower screening uptake can be explained by several plausible reasons. Tobacco-using women often experience stigma, self-blame and fear of judgment by healthcare providers, which discourages them from oral examinations [54, 55]. Various forms of tobacco use among women are frequently normalised within households, reducing perceived susceptibility and need for screening [56]. Health-system barriers such as limited counselling, lack of women-centred tobacco cessation support and inadequate provider engagement further limit uptake [57–59].

Strengthening screening would therefore require a gender-responsive approach. Community-based studies demonstrate that integrating visual oral examinations into routine primary care, engaging frontline healthcare workers for household-level motivation, and utilising patient-navigation systems can substantially enhance early detection. Indian evidence consistently demonstrates that screen-positive women are far more likely to complete diagnostic confirmation and initiate treatment when accompanied by a patient navigator or a trusted community member [60]. Such ‘known faces’ help address fear, stigma, low autonomy and logistical barriers that commonly prevent women from following through with referrals. Experiences from breast and cervical cancer programmes in Tamil Nadu and Kerala confirm that navigator-supported pathways reduce loss to follow-up and improve treatment compliance. Tailored communication and culturally sensitive counselling further enhance uptake and linkage to care.

The study has several strengths and some limitations that warrant consideration**.** A major strength lies in its use of a nationally representative dataset with a large and diverse sample size of 348,000 women. The dataset allows for an in-depth examination of disparities across geographic, socioeconomic and demographic divides using PROGRESS variables. Moreover, the integration of geospatial analysis and decomposition methods provides insight into the key drivers of gender inequities and urban-rural differences. However, the cross-sectional design of NFHS-5 limits the ability to make causal inferences and does not permit the establishment of a temporal relationship between exposure variables and screening behaviours. A key limitation of this study is that NFHS-5 collects cancer-screening information only for women aged 15–49 years; therefore, women aged ≥50 years, who also fall within the recommended age range for oral cancer screening under national guidelines, are not represented, potentially leading to an underestimation of true population-level screening coverage. Additionally, the reliance on self-reported data may have introduced recall and/or social desirability bias, particularly in sensitive domains like tobacco utilisation or cancer screening. While efforts were made during survey administration to minimise reporting inaccuracies, these biases cannot be ruled out. Prior engagements in programmatic evaluations and NCD surveillance inform us of the structural challenges underlying low screening uptake [61–63]. These positional insights underscore the need for grounded, culturally relevant and community-centered interventions that are tailored to specific needs.

In conclusion, the poor uptake of oral cancer screening among Indian women reflects deeper governance and systemic issues. National and subnational level policies must embrace decentralised and equity-responsive strategies, prioritising regions with low baseline coverage and high risk of oral cancer. Geographically embedded disparities also necessitate region- or state-specific investments, including mobile outreach, culturally appropriate health promotion campaigns and capacity building of healthcare workers in the identification and reporting of oral cancers in women. Addressing the identified inequities and ensuring diversity and inclusion in program implementation will be key to oral cancer control in India.

## Conflicts of interest

Authors have no conflicts of interest to declare.

## Funding

No funding or financial grant was received to conduct the present study.

## Authors’ contribution

NK, PH, YK and SR contributed to the conception and design of the study. NK, PH, GSJ and GB contributed to the analysis plan. PH, YK and SR contributed to the analysis and interpretation of data. NK, PH and GSJ drafted the manuscript. RS, ST and MG helped write the manuscript’s discussion. All authors reviewed the manuscript. All authors have approved the final version of the manuscript for submission.

## Ethics

The NFHS-5 received ethical approval from the International Institute for Population Sciences (IIPS), Mumbai (2019–21). It was also reviewed by the ICF International Review Board (IRB), which approved it ethically. After receiving complete information about the goal and methodology of the survey, the respondents signed to confirm their agreement. Interviews were conducted only after receiving each participant’s informed consent.

## Data availability

The study utilises data from a published summary of the NFHS-5, which is publicly accessible and can be obtained by registering at https://dhsprogram.com/Countries/CountryMain.cfm?ctry_id=57&c=India. The corresponding author can provide the processed data upon reasonable request.

## Consent for publication

Not applicable.

## Clinical trial number

Not applicable (this is a cross-sectional study).

## Figures and Tables

**Table 1.** Self-reported oral cancer screening by women who participated in the fifth National Family Health Survey, India (2019–21).

Variables	Overall sample N = 348,882			Urban N = 89,228			Rural N = 259,654		
	Unweighted frequency	Weighted proportions (95% CI)	p-value	Unweighted frequency	Weighted proportions (95% CI)	p-value	Unweighted frequency	Weighted proportions (95% CI)	p-value
Overall	*N* = 2,252	0.87 (0.84–0.90)	-	*N* = 749	1.08 (1.04–1.12)	-	*N* = 1,503	0.77 (0.74–0.80)	-
Demographic and socio-economic factors									
Age-group in years									
30–34	549	0.74 (0.71–0.77)	<0.001	179	0.93 (0.9–0.96)	0.015	370	0.65 (0.62–0.68)	0.012
35–39	588	0.85 (0.82–0.88)		187	1.07 (1.04–1.09)		401	0.74 (0.71–0.77)	
40–44	529	0.90 (0.86–0.93)		187	1.02 (0.99–1.06)		342	0.83 (0.81–0.86)	
45–49	586	1.02 (0.99–1.06)		196	1.34 (1.31–1.38)		390	0.87 (0.84–0.89)	
Religion									
Hindu	1,749	0.86 (0.83–0.89)	<0.001	581	1.09 (1.06–1.12)	0.002	1,168	0.75 (0.72–0.78)	<0.001
Muslim	220	0.61 (0.57–0.64)		77	0.64 (0.61–0.67)		143	0.59 (0.56–0.62)	
Christian	210	2.34 (2.31–2.36)		59	2.42 (2.38–2.45)		151	2.31 (2.27–2.33)	
Others	73	0.96 (0.93–0.98)		32	1.91 (1.88–1.94)		41	0.48 (0.45–0.51)	
Social caste									
Schedule caste	395	0.84 (0.81–0.87)	<0.001	126	1.02 (0.99–1.05)	0.028	269	0.76 (0.73–0.79)	<0.001
Schedule tribe	247	0.44 (0.41–0.47)		57	0.82 (0.79–0.85)		190	0.37 (0.34–0.41)	
Other backward castes	986	0.97 (0.94–1.01)		331	1.19 (1.16–1.21)		655	0.86 (0.83–0.89)	
Others	624	0.89 (0.86–0.92)		235	1.01 (0.98–1.04)		389	0.79 (0.76–0.82)	
Wealth quintile									
Poorest	241	0.37 (0.34–0.41)	<0.001	8	0.37 (0.34–0.39)	0.021	233	0.37 (0.34–0.41)	<0.001
Poorer	389	0.65 (0.62–0.68)		45	0.65 (0.62–0.68)		344	0.65 (0.62–0.68)	
Middle	550	1.02 (0.99–1.05)		117	1.02 (0.99–1.05)		433	1.02 (0.99–1.05)	
Richer	587	1.14 (1.11–1.17)		237	1.13 (1.09–1.16)		350	1.15 (1.12–1.18)	
Richest	485	1.09 (1.06–1.12)		342	1.18 (1.15–1.21)		143	0.86 (0.83–0.89)	
Education									
Illiterate	725	0.77 (0.74–0.81)	<0.001	129	0.99 (0.96–1.01)	0.199	596	0.72 (0.69–0.75)	0.038
Primary	335	0.93 (0.9–0.96)		96	1.34 (1.31–1.37)		239	0.79 (0.76–0.82)	
Secondary	946	0.92 (0.89–0.95)		357	1.06 (1.03–1.09)		589	0.81 (0.78–0.84)	
Higher Secondary	246	0.97 (0.94–0.99)		167	1.06 (1.02–1.09)		79	0.78 (0.75–0.81)	
Gender of head of household									
Male	1,871	0.86 (0.83–0.89)	0.851	615	1.05 (1.02–1.08)	0.855	1,256	0.76 (0.73–0.79)	0.981
Female	381	0.95 (0.92–0.98)		134	1.24 (1.21–1.26)		247	0.81 (0.78–0.84)	
Marital status									
Married	2,026	0.86 (0.83–0.89)	0.218	658	1.08 (1.04–1.11)	0.234	1,368	0.75 (0.72–0.78)	0.761
Others	226	1.02 (0.98–1.05)		91	1.12 (1.09–1.15)		135	0.96 (0.93–0.99)	
Health insurance									
No	1,262	0.64 (0.61–0.67)	<0.001	433	0.85 (0.81–0.88)	<0.001	829	0.53 (0.49–0.56)	<0.001
Yes	990	1.30 (1.27–1.33)		316	1.62 (1.59–1.65)		674	1.17 (1.14–1.21)	
Region									
North	233	0.31 (0.27–0.34)	<0.001	77	0.41 (0.38–0.44)	<0.001	156	0.24 (0.21–0.27)	<0.001
Central	352	0.51 (0.48–0.55		95	0.63 (0.61–0.66)		257	0.47 (0.44–0.49)	
East	103	0.18 (0.15–0.21)		19	0.16 (0.13–0.19)		84	0.19 (0.16–0.22)	
Northeast	202	0.33 (0.31–0.36)		86	0.71 (0.68–0.74)		116	0.23 (0.19–0.26)	
West	186	0.86 (0.83–0.89)		78	1.11 (1.08–1.14)		108	0.67 (0.64–0.71)	
South	1,176	2.31 (2.27–2.34)		394	2.29 (2.26–2.32)		782	2.33 (2.29–2.36)	
Health related									
BMI									
Underweight	181	0.63 (0.61–0.66)	<0.001	28	0.91 (0.88–0.94)	<0.001	153	0.58 (0.55–0.61)	<0.001
Normal	749	0.68 (0.65–0.71)		205	0.87 (0.84–0.91)		544	0.61 (0.58–0.64)	
Overweight	371	0.73 (0.71–0.76)		129	0.94 (0.91–0.97)		242	0.62 (0.59–0.65)	
Obesity	951	1.23 (1.21–1.26)		387	1.31 (1.28–1.34)		564	1.17 (1.14–1.20)	
Diabetes									
No	2,094	0.83 (0.81–0.86)	<0.001	681	1.01 (0.98–1.04)	<0.001	1,413	0.74 (0.71–0.77)	<0.001
Yes	137	2.24 (2.21–2.27)		62	2.75 (2.72–2.78)		75	1.79 (1.77–1.83)	
Don’t know	21	0.44 (0.41–0.47)		6	0.51 (0.48–0.54)		15	0.42 (0.39–0.45)	
Hypertension									
No	2,022	0.85 (0.82–0.88)	<0.001	662	1.06 (1.03–1.09)	0.026	662	0.75 (0.72–0.78)	<0.001
Yes	219	1.19 (1.16–1.22)		82	1.37 (1.34–1.41)		82	1.08 (1.05–1.11)	
Don’t know	11	0.43 (0.41–0.46)	5	0.84 (0.81–0.87)	5	0.31 (0.28–0.34)
Behavioural factors									
Eat fruits									
Never	27	0.61 (0.58–0.64)	<0.001	11	1.81 (1.78–1.84)	<0.001	16	0.29 (0.26–0.32)	<0.001
Daily	356	1.15 (1.12–1.18)		189	1.24 (1.21–1.27)		167	1.03 (0.99–1.06)	
Weekly	913	0.96 (0.93–0.99)		340	1.13 (1.11–1.16)		573	0.85 (0.82–0.88)	
Occasionally	956	0.75 (0.72–0.78)		209	0.91 (0.88–0.94)		747	0.70 (0.67–0.73)	
Eat fried food									
Never	129	0.99 (0.95–1.02)	0.234	49	1.42 (1.39–1.45)	0.058	80	0.79 (0.76–0.82)	0.364
Daily	194	0.62 (0.59–0.65)		76	0.67 (0.64–0.71)		118	0.59 (0.56–0.62)	
Weekly	717	0.89 (0.85–0.92)		251	1.00 (0.97–1.03)		466	0.83 (0.79–0.86)	
Occasionally	1,212	0.88 (0.85–0.91)		373	1.17 (1.14–1.2)		839	0.75 (0.72–0.78)	
Chew tobacco									
No	2,217	0.88 (0.85–0.91)	0.003	741	1.10 (1.07–1.13)	0.63	1,476	0.77 (0.74–0.80)	0.026
Yes	35	0.34 (0.30–0.37)		8	0.18 (0.15–0.21)		27	0.39 (0.36–0.42)	
Smoking									
No	2,077	0.9 (0.87–0.93)	0.015	703	1.11 (1.08–1.14)	0.852	1,374	0.79 (0.76–0.82)	0.032
Yes	175	0.43 (0.39–0.46)		46	0.42 (0.39–0.45)		129	0.43 (0.4–0.46)	
Alcohol consumption									
No	2,203	0.88 (0.85–0.91)	0.100	743	1.09 (1.06–1.12)	0.459	1,460	0.77 (0.74–0.81)	0.328
Yes	49	0.53 (0.49–0.56)		6	0.46 (0.43–0.49)		43	0.54 (0.51–0.57)	
Exposure of media									
No	415	0.54 (0.51–0.57)	<0.001	53	0.70 (0.67–0.73)	0.011	362	0.52 (0.49–0.55)	<0.001
Yes	1,837	0.99 (0.96–1.02)		696	1.13 (1.10–1.16)		1,141	0.90 (0.87–0.93)	

**Table 2.** Factors contributing to differences in urban-rural self-reported screening coverage among women participants of NFHS-5.

Factors	Urban (A) - Rural (B)				
	Difference in the composition (endowment) (E)		Differences in the effect of characteristics (coefficient) (C)		
	Coefficient (%) (95% CI)	%	Coefficient (%) (95% CI)	%	
Demographic and socio-economic factors					
Age-group in years					
30–34	Ref.	-	Ref.	-	
35–39	0 (0 to 0.001)	0.00	0.001 (−0.089 to 0.089 )	0.05	
40–44	−0.001 (−0.002 to 0.001)	−0.20	−0.036 (−0.122 to 0.05 )	−11.35	
45–49	0 (−0.002 to 0.001)	−0.08	0.015 (−0.068 to 0.097 )	4.70	
Religion					
Hindu	Ref.	-	Ref.	-	
Muslim	−0.02 (−0.033 to −0.008)*	−6.41	−0.048 (−0.124 to 0.027 )	−15.16	
Christian	0.001 (−0.001 to 0.002)	0.27	−0.005 (−0.019 to 0.009 )	−1.47	
Other religious minorities	0.001 (0.001 to 0.001)*	0.03	0.016 (−0.016 to 0.048 )	5.09	
Caste					
Schedule caste	Ref.	-	Ref.	-	
Schedule tribe	0.007 (−0.028 to 0.042)	2.24	0.03 (−0.054 to 0.115 )	9.60	
Other backward castes	0 (0 to 0)	−0.01	−0.019 (−0.156 to 0.117 )	−6.12	
General caste	0.038 (0.01 to 0.065)*	11.87	0.01 (−0.077 to 0.097 )	3.15	
Education					
Illiterate	Ref.	-	Ref.	-	
Primary	−0.006 (−0.018 to 0.006)	−1.92	0.042 (−0.045 to 0.129 )	13.24	
Secondary	−0.021 (−0.047 to 0.006)	−6.51	−0.002 (−0.106 to 0.102 )	−0.62	
Higher Secondary	−0.029 (−0.067 to 0.009)	−9.17	0.007 (−0.017 to 0.03 )	2.09	
Gender of head of household					
Male	Ref.	-	Ref.	-	
Female	0 (−0.001 to 0)	−0.08	0.023 (−0.043 to 0.088 )	7.19	
Marital status					
Not in union, widowed, divorced/separated	Ref.	-	Ref.		-
Married	−0.003 (−0.008 to 0.003)	−0.81	0.162 (−0.289 to 0.613 )		51.18
Health insurance					
No	Ref.	-	Ref.		-
Yes	−0.019 (−0.028 to −0.01)*	−5.91	−0.018 (−0.095 to 0.06 )		−5.53
Region					
North	Ref.	-	Ref.		-
Central	−0.038 (−0.06 to −0.016)*	−12.11	−0.023 (−0.128 to 0.083 )		−7.23
East	0.041 (0.011 to 0.071)*	12.85	−0.135 (−0.41 to 0.14 )		−42.53
Northeast	−0.015 (−0.023 to −0.006)*	−4.69	0.042 (−0.017 to 0.101 )		13.28
West	0.065 (0.035 to 0.095)*	20.41	−0.013 (−0.071 to 0.044 )		−4.17
South	0.107 (0.073 to 0.140)*	33.73	−0.074 (−0.169 to 0.021 )		−23.41
Health related factors					
BMI					
Underweight (<18.5)	Ref.	-	Ref.		-
Normal (18.5–22.9)	0.001 (−0.044 to 0.045)	0.17	−0.014 (−0.266 to 0.238 )		−4.37
Overweight (23.0–24.9)	0 (−0.007 to 0.007)	−0.04	0.012 (−0.097 to 0.121 )		3.72
Obesity (>25.0)	0.021 (−0.048 to 0.089)	6.50	−0.046 (−0.219 to 0.126 )		−14.66
Diabetes					
No	Ref.	-	Ref.		-
Yes	0.011 (0.005 to 0.018)*	3.57	0.008 (−0.007 to 0.024 )		2.66
Don’t know	0.002 (−0.002 to 0.005)	0.50	−0.006 (−0.023 to 0.012 )		−1.86
Hypertension					
No	Ref.	-	Ref.		-
Yes	0.001 (−0.002 to 0.004)	0.32	−0.014 (−0.045 to 0.017)		−4.45
Don’t know	−0.001 (−0.004 to 0.002)	−0.36	0.007 (−0.008 to 0.022)		2.18
Behavioural factors					
Eat fruits					
Never	Ref.	-	Ref.		-
Daily	−0.048 (−0.127 to 0.032)	−14.98	−0.09 (−0.229 to 0.049 )		−28.39
Weekly	−0.041 (−0.107 to 0.025)	−12.85	−0.382 (−0.969 to 0.206 )		−120.34
Occasionally	0.098 (−0.039 to 0.235)	30.92	−0.758 (−1.866 to 0.351 )		−238.86
Eat fried food					
Never	Ref.	−	Ref.		-
Daily	−0.001 (−0.003 to 0.001)	−0.42	−0.031 (−0.09 to 0.027 )		−9.90
Weekly	−0.001 (−0.01 to 0.007)	−0.46	−0.115 (−0.329 to 0.098 )		−36.35
Occasionally	0 (−0.008 to 0.007)	−0.03	−0.019 (−0.281 to 0.242 )		−6.11
Chew tobacco					
No	Ref.	-	Ref.		-
Yes	0.007 (0 to 0.014)	2.22	−0.019 (−0.055 to 0.017 )		−5.99
Smoking					
No	Ref.	-	Ref.		-
Yes	0.01 (−0.008 to 0.028)	3.17	−0.014 (−0.069 to 0.042 )		−4.41
Alcohol consumption					
No	Ref.	-	Ref.		-
Yes	0.007 (−0.001 to 0.015)	2.16	−0.009 (−0.029 to 0.011 )		−2.79
Exposure to Media					
Yes	Ref.	-	Ref.		-
No	0.012 (−0.051 to 0.075)	3.87	0.186 (−0.197 to 0.569 )		58.62
Overall	0.183 (0.094 to 0.273)*	57.76	0.134 (0.007 to 0.261)*		42.24
Overall difference (A−B) = (E+C)	0.317 (0.196 to 0.261)*	-	-		-

## Supplementary Table S1. Distribution of various factors as per coverage of oral cancer screening among Indian women aged 30–49years.

**Table:**

Variables	Screening for oral cancer					
	Overall sample		Urban		Rural	
	*N* = 348,882	Weighted proportion	*N* = 89,228	Weighted proportion	*N* = 259,654	Weighted proportion
Overall screening coverage		0.87%		1.08%		0.77%
Demographic and socio-economic factors						
Age-group in years						
30–34	27.54	0.74*	27.34	0.93*	27.64	0.65*
35–39	26.69	0.85*	26.84	1.07*	26.62	0.74*
40–44	22.43	0.90*	22.87	1.02*	22.21	0.83*
45–49	23.34	1.02*	22.95	1.34*	23.54	0.87*
Religion						
Hindu	82.49	0.86*	78.91	1.09*	84.29	0.75*
Muslim	12.03	0.61*	15.29	0.64*	10.40	0.59*
Christian	2.57	2.34*	2.89	2.42*	2.41	2.30*
Others	2.91	0.96*	2.91	1.91*	2.90	0.48*
Caste						
Schedule caste	21.29	0.84*	18.71	1.02*	22.59	0.76*
Schedule tribe	9.16	0.44*	4.08	0.82*	11.71	0.37*
OBC	42.92	0.97*	42.81	1.19*	42.97	0.86*
Others	26.63	0.89*	34.40	1.01*	22.73	0.79*
Wealth index						
Poorest	18.01	0.37*	2.68	0.37*	25.72	0.37*
Poorer	19.34	0.65*	6.70	0.65*	25.69	0.65*
Middle	20.65	1.02*	15.57	1.02*	23.20	1.02*
Richer	21.15	1.14*	29.70	1.13*	16.85	1.15*
Richest	20.84	1.09*	45.35	1.18*	8.53	0.86*
Education						
Illiterate	35.60	0.77*	19.54	0.99	43.67	0.72*
Primary	15.58	0.93*	12.42	1.34	17.17	0.79*
Secondary	38.60	0.92*	47.61	1.06	34.07	0.81*
Higher secondary	10.22	0.97*	20.43	1.06	5.09	0.78*
Gender of HOH						
Male	83.46	0.86	83.64	1.05	83.37	0.76
Female	16.54	0.95	16.36	1.24	16.63	0.81
Marital status						
Married	91.11	0.86	89.85	1.08	91.74	0.75
Others	8.89	1.02	10.15	1.12	8.26	0.96
Health insurance						
No	65.57	0.64*	69.57	0.85*	63.56	0.53*
Yes	34.43	1.30*	30.43	1.62*	36.44	1.17*
Region						
North	20.04	0.31*	15.18	0.41*	13.03	0.24*
Central	21.24	0.51*	17.61	0.63*	24.71	0.47*
East	15.64	0.18*	16.58	0.16*	24.94	0.19*
Northeast	14.98	0.33*	2.40	0.71*	4.58	0.23*
West	10.60	0.86*	20.01	1.11*	12.26	0.67*
South	17.49	2.31*	28.22	2.29*	20.48	2.33*
Health related factors						
BMI						
Underweight (<18.5)	10.26	0.63*	5.23	0.91*	12.79	0.58*
Normal (18.5–22.9)	38.61	0.68*	31.55	0.87*	42.16	0.61*
Overweight (23.0–24.9)	17.51	0.73*	18.58	0.94*	16.97	0.62*
Obesity (>25.0)	33.62	1.23*	44.63	1.31*	28.08	1.17*
Diabetes						
No	95.68	0.83*	94.77	1.01*	96.13	0.74*
Yes	3.19	2.24*	4.47	2.75*	2.56	1.80*
Don’t know	1.13	0.44*	0.77	0.51*	1.31	0.42*
Hypertension						
No	91.76	0.85*	91.12	1.06*	92.08	0.75*
Yes	7.53	1.19*	8.42	1.37*	7.09	1.08*
Don’t know	0.71	0.43*	0.47	0.84*	0.83	0.31*
Behavioural factors						
Eat fruits						
Never	1.87	0.61*	1.20	1.81*	2.20	0.29*
Daily	11.93	1.15*	20.17	1.24*	7.78	1.03*
Weekly	36.62	0.96*	43.58	1.13*	33.12	0.85*
Occasionally	49.59	0.75*	35.05	0.91*	56.89	0.70*
Eat fried food						
Never	4.87	0.99	4.44	1.42	5.08	0.79
Daily	7.31	0.62	7.67	0.67	7.13	0.59
Weekly	34.30	0.89	36.11	1.00	33.39	0.83
Occasionally	53.52	0.88	51.78	1.17	54.40	0.75
Chew tobacco						
No	98.01	0.88*	98.66	1.10	97.69	0.77*
Yes	1.99	0.34*	1.34	0.18	2.31	0.39*
Smoking						
No	93.79	0.90*	96.29	1.11	92.53	0.79*
Yes	6.21	0.43*	3.71	0.42	7.47	0.43*
Alcohol consumption						
No	98.90	0.88	99.57	1.09	98.57	0.77
Yes	1.10	0.53	0.43	0.46	1.43	0.54
Exposure of Media						
No	26.58	0.54*	10.07	0.70*	34.88	0.52*
Yes	73.42	0.99*	89.93	1.13*	65.12	0.90*

*Chi-square *p*-value < 0.05.

## Supplementary Table S2. Sub-national coverage of oral cancer screening among Indian women 15–49 years.

**Table:**

States/Union territories	Women		
	Overall	Urban	Rural
India	0.87	1.08	0.77
North	0.31	0.41	0.24
Chandigarh	0.45	0.45	0.00
Delhi	0.91	0.90	1.14
Haryana	0.29	0.31	0.28
Himachal Pradesh	0.26	0.00	0.30
Jammu & Kashmir	0.66	0.05	0.90
Ladakh	0.18	0.00	0.22
Punjab	0.22	0.24	0.20
Rajasthan	0.14	0.24	0.10
Uttarakhand	0.34	0.00	0.49
Central	0.51	0.63	0.47
Chhattisgarh	0.22	0.44	0.15
Madhya Pradesh	0.66	1.01	0.53
Uttar Pradesh	0.50	0.50	0.50
East	0.18	0.16	0.19
Bihar	0.27	0.37	0.25
Jharkhand	0.16	0.17	0.15
Odisha	0.26	0.14	0.28
West Bengal	0.09	0.07	0.11
North East	0.33	0.71	0.23
Arunachal Pradesh	0.54	0.96	0.46
Assam	0.22	0.45	0.17
Manipur	0.99	2.00	0.32
Meghalaya	0.44	0.42	0.45
Mizoram	0.81	1.22	0.26
Nagaland	0.37	0.55	0.28
Sikkim	0.60	0.97	0.41
Tripura	0.54	0.71	0.46
West	0.86	1.11	0.67
Dadra & Nagar Haveli and Daman & Diu	0.18	0.38	0.00
Goa	0.56	0.80	0.22
Gujarat	0.19	0.18	0.19
Maharashtra	1.22	1.57	0.92
South	2.31	2.29	2.33
Andaman & Nicobar Islands	10.32	10.32	10.32
Andhra Pradesh	7.46	8.44	7.02
Karnataka	0.38	0.35	0.40
Kerala	0.66	0.82	0.52
Lakshadweep	0.28	0.34	0.00
Puducherry	1.52	2.04	0.41
Tamil Nadu	1.18	1.38	1.01
Telangana	2.48	3.19	2.11

## References

[1] Kocarnik JM, Compton K, Dean FE (2022). Cancer incidence, mortality, years of life lost, years lived with disability, and disability-adjusted life years for 29 cancer groups from 2010 to. JAMA Oncol.

[2] Ferrari AJ, Santomauro DF, Aali A (2024). Global incidence, prevalence, years lived with disability (YLDs), disability-adjusted life-years (DALYs), and healthy life expectancy (HALE) for 371 diseases and injuries in 204 countries and territories and 811 subnational locations, 1990–2021: a systema. Lancet.

[3] Johnson S, McDonald JT, Corsten M (2012). Oral cancer screening and socioeconomic status. J Otolaryngol Head Neck Surg.

[4] Bray F, Laversanne M, Sung H (2024). Global cancer statistics 2022: gLOBOCAN estimates of incidence and mortality worldwide for 36 cancers in 185 countries. CA Cancer J For Clinicians.

[5] Coelho KR (2012). Challenges of the oral cancer burden in India. J Cancer Epidemiol.

[6] Ferlay J, Colombet M, Soerjomataram I (2021). Cancer statistics for the year 2020: an overview. Int J Cancer.

[7] Mathur P, Sathishkumar K, Das P (2025). Global burden of 292 causes of death in 204 countries and territories and 660 subnational locations , 1990 – 2023 : a systematic analysis for the Global Burden of Disease Study 2023 Summary Background. JAMA Netw Open.

[8] Dhane AS (2024). Understanding oral cancer disparities in India: exploring socioeconomic and demographic factors impacting access to care and outcomes. Oral Oncol Rep.

[9] Gupta B, Bray F, Kumar N (2017). Associations between oral hygiene habits, diet, tobacco and alcohol and risk of oral cancer: a case–control study from India. Cancer Epidemiol.

[10] Kretzler B, König HH, Brandt L (2022). Religious denomination, religiosity, religious attendance, and cancer prevention. A systematic review. Risk Manag Healthc Policy.

[11] Sankaranarayanan R, Ramadas K, Amarasinghe H (2015). Oral cancer: prevention, early detection, and treatment. Disease Control Priorities, Third Edition (Volume 3): Cancer.

[12] Ministry of Health and Family Welfare; Government of India (2016). Operational framework on the managment of common cancers [Internet] Vol. https://mohfw.gov.in/sites/default/files/Operational.

[13] Sankaranarayanan R, Ramadas K, Thara S (2013). Long term effect of visual screening on oral cancer incidence and mortality in a randomized trial in Kerala, India. Oral Oncol.

[14] International Institute for Population Sciences (2021). National family health survey (NFHS-5) India 2019–21 [Internet]. Demographic and health surveys. https://dhsprogram.com/methodology/survey/survey-display-541.cfm.

[15] Global Adult Tobacco Survey (GATS) (2017). Public use data file, India.

[16] Tata Institute of Social Sciences, Ministry of Health and Family Welfare Government of India (2010). Global adult tobacco survey India 2009–2010 [Internet]. https://www.who.int/tobacco/surveillance/survey/gats/ind/en/.

[17] Goyal LD, Verma M, Garg P (2022). Variations in the patterns of tobacco usage among Indian females - findings from the global adult tobacco survey India. BMC Womens Health.

[18] Kumar A (2023). The transformation of The Indian Healthcare System. Cureus.

[19] Mishra GA, Shaikh HA, Pimple SA (2021). Determinants of compliance to population-based oral cancer screening program among low socioeconomic women in Mumbai, India. Indian J Community Med.

[20] Changkun Z, Bishwajit G, Ji L (2022). Sociodemographic correlates of cervix, breast and oral cancer screening among Indian women. PLoS One.

[21] The Demographic Health Survey Program (2020). DHS survey design: frequently asked questions what factors determine sample size? [Internet]. https://dhsprogram.com/pubs/pdf/SAR15/SAR15.pdf.

[22] The Demographic Health Surveys Program (2024). DHS methodology [Internet]. https://dhsprogram.com/methodology/survey-types/DHS-Methodology.cfm.

[23] Ministry of Health and Family Welfare; Govt. of India (2016). Population based screening of common non-communicable diseases [Internet]. Training module for staff nurses. https://ab-hwc.nhp.gov.in/download/document/829bf86e304132dfae9962855c57dcd8.pdf.

[24] Garg P, Krishnamoorthy Y, Halder P (2025). Urban-rural disparities in cervical cancer screening among Indian women between 30–49 years: a geospatial and decomposition analysis using a nationally representative survey. BMC Cancer.

[25] Banerjee S (2021). Determinants of rural-urban differential in healthcare utilization among the elderly population in India. BMC Public Health.

[26] Shruti T, Khanna D, Khan A (2023). Status and determinants of early detection of oral premalignant and malignant lesions in India. Cancer Control.

[27] Res M, Centre N, Aids N (2012). Validity of self-reported morbidity. Indian J Med Res.

[28] Tugwell P, Petticrew M, Kristjansson E (2010). Assessing equity in systematic reviews: realising the recommendations of the commission on social determinants of health. BMJ.

[29] Anselin L, Syabri I, Kho Y (2006). GeoDa : an introduction to spatial data analysis. Geogr Anal.

[30] Powers D, Yoshioka H, Yun MS (2011). Mvdcmp: multivariate decomposition for nonlinear response models. Stata J.

[31] Karran EL, Cashin AG, Barker T (2023). Using PROGRESS-plus to identify current approaches to the collection and reporting of equity-relevant data: a scoping review. J Clin Epidemiol.

[32] Mayo M, Potugari B, Bzeih R (2021). Cancer screening during the COVID-19 pandemic: a systematic review and meta-analysis. Mayo Clin Proc Innov Qual Outcomes.

[33] Luciani S, Caixeta R, Chavez C (2023). What is the NCD service capacity and disruptions due to COVID-19? Results from the WHO non-communicable disease country capacity survey in the Americas region. BMJ Open.

[34] United Nations (2021). Tackling inequalities in public service coverage to “build forward better” for the rural poor - policy brief [Internet]. https://unsceb.org/sites/default/files/2021-12/HLCP.

[35] Sen S, Khan PK, Wadasadawala T (2022). Socio-economic and regional variation in breast and cervical cancer screening among Indian women of reproductive age: a study from National Family Health Survey, 2019–21. BMC Cancer.

[36] Sinha A, Kumar Sedai A, Bahadur Rahut D (2024). Well-being costs of unpaid care: gendered evidence from a contextualized time-use survey in India. World Dev.

[37] Kundra S, Sreen N, Dwivedi R (2023). Impact of work from home and family support on Indian women’s work productivity during COVID-19. Vikalpa J Decis Makers.

[38] Singh SK, Chauhan K, Puri P (2023). Chronic non-communicable disease burden among reproductive-age women in India: evidence from recent demographic and health survey. BMC Women's Health.

[39] Idris IB, Hamis AA, Bukhori ABM (2023). Women’s autonomy in healthcare decision making: a systematic review. BMC Womens Health.

[40] Thapa R, Van Teijlingen E, Regmi PR (2021). Caste exclusion and health discrimination in South Asia: a systematic review. Asia Pacific J Public Heal.

[41] Ahmed S, Mahapatro S (2023). Inequality in healthcare access at the intersection of caste and gender. Contemp Voice Dalit.

[42] Siva N, Mohanty K, Rath R (2025). A systematic scoping review of health-seeking behavior and healthcare utilization in tribal communities of Odisha, India: concentration on maternal and child health. BMC Public Health.

[43] Rogers ES, Wallace LS, Weiss BD (2006). Misperceptions of medical understanding in low-literacy patients: implications for cancer prevention. Cancer Control.

[44] ecancer (2023). Survey reveals widespread confusion and misconceptions about ovarian cancer screening among a majority of women -ecancer [Internet]. News.

[45] Karanth S, Mistry S, Wheeler M (2024). Persistent poverty disparities in incidence and outcomes among oral and pharynx cancer patients. Cancer Causes Control.

[46] Ismail AI, Jedele JM, Lim S (2012). A marketing campaign to promote screening for oral cancer. J Am Dent Assoc.

[47] Schliemann D, Su TT, Paramasivam D (2019). Effectiveness of mass and small media campaigns to improve cancer awareness and screening rates in Asia: a systematic review. J Glob Oncol.

[48] Gopika MG, Prabhu PR, Thulaseedharan JV (2022). Status of cancer screening in India: an alarm signal from the National Family Health Survey (NFHS-5). J Fam Med Prim Care.

[49] Ministry of Health and Family Welfar (2019). National programme for prevention and control of cancer, diabetes, cardiovascular diseases and stroke (NPCDCS) [Internet]. https://main.mohfw.gov.in/Major-Programmes/non-communicable-diseases-injury-trauma/Non-Communicable-Disease-II/National-Programme-for-Prevention-and-Control-of-Cancer-Diabetes-Cardiovascular-diseases-and-Stroke-NPCDCS.

[50] Ministry of Health and Family Welfare (2023). National programme for prevention and control of non-communicable diseases (2023–2030) [Internet]. Operational guidelines. https://pib.gov.in/PressReleseDetail.aspx?PRID=1924730.

[51] Bernard M, Löbner M, Lordick F (2022). Cancer prevention in females with and without obesity: does perceived and internalised weight bias determine cancer prevention behaviour?. BMC Womens Health.

[52] Urbute A, Kjaer SK, Kesmodel US (2022). Women with obesity participate less in cervical cancer screening and are more likely to have unsatisfactory smears: results from a nationwide Danish cohort study. Prev Med (Baltim).

[53] Menon I, Kumar P, Rebello P (2025). Oral cancer awareness and screening practices in urban versus rural Indian population: web-based survey. Bioinformation.

[54] Goel S, Walia D, Kumar R (2024). The hidden crisis: health impacts of tobacco and nicotine products on Indian women. J Fam Med Prim Care.

[55] Kumar DV, Amandeep D, Rathee DS (2025). Barriers to treatment seeking among female smokers in rural community in north India; a qualitative study. J Popul Ther Clin Pharmacol.

[56] Jassem E (2014). The many faces of tobacco use among women. Med Sci Monit.

[57] Sultana S, Inungu J, Jahanfar S (2025). Barriers and facilitators of tobacco cessation interventions at the population and healthcare system levels: a systematic literature review. Int J Environ Res Public Health.

[58] Pollak KI, Arredondo EM, Yarnall KSH (2001). How do residents prioritize smoking cessation for young 'high-risk' women? factors associated with addressing smoking cessation. Prev Med (Baltim).

[59] Chaudhary J, Gupta E, Singh PK (2023). Tobacco exposure among antenatal women in India: challenges in tobacco screening & cessation counselling. Indian J Med Res.

[60] Sharma P, Khanna D, Pradhan S (2023). Community cancer screening at primary care level in Northern India: determinants and policy implications for cancer prevention. Fam Med Community Heal.

[61] Thakur J, Jeet G, Nangia R (2019). Non-communicable diseases risk factors and their determinants: a cross-sectional statewide STEPS survey, Haryana, North India. PLoS One.

[62] Thakur JS, Jeet G, Pal A (2016). Profile of risk factors for non-communicable diseases in Punjab, Northern India: results of a state-wide STEPS survey. PLoS One.

[63] Thakur J, Prinja S, Jeet G (2016). Costing of a state-wide population based cancer awareness and early detection campaign in a 2.67 million population of Punjab state in Northern India. Asian Pacific J Cancer Prevention.

